# Flavonoid-Rich *Cyperus esculentus* Extracts Disrupt Cellular and Metabolic Functions in *Staphylococcus aureus*

**DOI:** 10.3390/microorganisms14010260

**Published:** 2026-01-22

**Authors:** Yaning Zhang, Zhengdong Ma, Xuzhe Wang, Qilong Jiang, Xue Kang, Hongmei Gao

**Affiliations:** College of Animal Science and Technology, Shihezi University, Shihezi 832000, China; z2357678055@163.com (Y.Z.); 13119025107@163.com (Z.M.); 13909907594@163.com (Q.J.); z23576780550805@163.com (X.K.); zd031101@sina.com (H.G.)

**Keywords:** *Cyperus esculentus* L., synergy of plant extracts, *Staphylococcus aureus*, flavonoid, antibacterial mechanism

## Abstract

The escalating threat of antibiotic resistance, particularly from *Staphylococcus aureus* (*S. aureus*), has become a critical challenge in both public health and animal husbandry. The extensive use of conventional antibiotics in livestock production accelerates the emergence of resistant strains, heightening risks to food safety and human health. Although plant-derived bioactive compounds are increasingly recognized as promising alternatives to synthetic antimicrobials, the mechanisms underlying their efficacy—and the potential for synergistic action among different plant parts—remain poorly understood. In particular, the antibacterial interactions among extracts from different tissues of *Cyperus esculentus* L. (*C. esculentus*), a plant rich in flavonoids and phenolics, have yet to be systematically evaluated. Here, we investigated the antibacterial properties and mechanisms of ethanol extracts from the tubers, stems–leaves and their mixture of *C. esculentus* against *S. aureus*. Using Oxford cup diffusion assays, scanning electron microscopy (SEM), bacterial growth kinetics, and untargeted metabolomics, we assessed both phenotypic inhibition and metabolic disruption. The mixed extract exhibited the strongest antibacterial effect, producing a 26.15 mm inhibition zone—approximately 7% greater than that of single-part extracts—and induced cell wall rupture and disintegration as observed by SEM. Growth curve analyses revealed time-dependent bacterial suppression, while metabolomic profiling identified 845 differential metabolites, indicating disturbances in amino acid, lipid, and nucleotide metabolism. Flavonoids such as acacetin, diosmetin, naringenin, and silybin A were identified as principal active compounds contributing to these effects.

## 1. Introduction

The overreliance on antibiotics in modern animal husbandry has led to the rapid emergence and dissemination of antibiotic-resistant bacterial strains, posing a serious and multifaceted threat to global public health [1]. This phenomenon not only impairs animal health and reduces agricultural productivity but also promotes the transmission of antibiotic-resistant bacteria via the food chain and environmental pathways, thereby elevating the risk of human exposure and subsequent infection. Among these resistant pathogens, *Staphylococcus aureus* (*S. aureus*) is a particularly notable opportunistic bacterium of high concern. Ubiquitously present on the skin and mucosal surfaces of livestock and poultry, *S. aureus* has developed formidable multidrug resistance, complicating its control in both veterinary and human health contexts [2,3]. Its ability to persist in perishable food products—such as dairy and meat—further amplifies its threat by contributing to spoilage, shortening shelf life, and elevating the incidence of foodborne illness [4]. Moreover, the pathogen’s potential for zoonotic transmission, via direct or indirect contact, can lead to a range of severe infections in humans, including cellulitis, pneumonia, and septicemia [5]. Resistant *S. aureus* strains necessitate innovations beyond conventional antibiotics, while safeguarding animal health, supporting agricultural sustainability, optimizing food preservation, and curbing human infections. In this regard, the exploration of natural, safe, and environmentally compatible antimicrobial agents has gained considerable momentum [6]. Plant-derived bioactive compounds, in particular, have drawn growing attention due to their structural diversity, ecological renewability, and potential for multi-target antimicrobial action. The multi-target antibacterial strategy exerts effects on multiple key bacterial targets simultaneously (e.g., cell wall, cell membrane, DNA, and metabolic processes), which can effectively eliminate bacteria and markedly delay the development of drug resistance. Its core evolutionary advantage lies in that bacteria have to acquire multiple independent and high-cost mutations to survive, thus greatly increasing the difficulty of their resistance evolution. Mixed extracts (such as plant extracts or traditional Chinese medicine formulae) mimic the “screening hypothesis” of chemical diversity in nature; their various bioactive components may jointly exert multi-target pressure through synergistic effects, thereby constructing an evolutionary defense line that is more difficult to be breached by single mutations.

Nucleotide biosynthesis forms a core metabolic pathway in *Staphylococcus aureus*, providing raw materials for nucleic acid synthesis and the energy currency ATP to sustain rapid bacterial proliferation. It is also deeply involved in key pathogenic processes, such as biofilm formation and the maintenance of drug resistance. For rapid proliferation, robust nucleotide metabolism is the cornerstone of bacterial replication and protein synthesis. Regarding biofilm formation, nucleotide metabolites are essential components of the biofilm matrix. The structural stability of biofilms is highly dependent on extracellular DNA (eDNA), which is directly derived from nucleotide polymerization. For the maintenance of drug resistance, the nucleotide biosynthetic pathway provides critical support for bacteria to withstand antibiotic stress. For example, antibiotics such as trimethoprim-sulfamethoxazole (TMP/SMX) exert bactericidal effects by interfering with folate metabolism, thereby inhibiting nucleotide synthesis. More importantly, decreased nucleotide metabolic activity is closely associated with bacteria entering a metabolically dormant persister state. In contrast, exogenous supplementation of nucleotide metabolites (e.g., adenosine, uracil) can activate the purine/pyrimidine salvage pathway, restore ATP levels, and resume protein synthesis in persister cells, thereby “awakening” their metabolism and resensitizing them to antibiotics. Thus, nucleotide biosynthesis acts as a core hub that integrates the growth, virulence, and adaptability of *Staphylococcus aureus*, providing a critical metabolic perspective for understanding its pathogenic mechanisms and developing novel antibacterial therapies.

Among these emerging candidates, *Cyperus esculentus* L. (*C. esculentus*) has recently attracted scientific interest not merely for its nutritional profile but for its promising biofunctional properties, including antimicrobial activity [7]. Its historical use as a food source, coupled with modern evidence suggesting bioactivity in its extracts, positions it as a viable resource in the search for natural antimicrobial agents [8]. Unlike many conventional crops, *C. esculentus* is notable for producing a diverse array of secondary metabolites with potential therapeutic relevance [9,10]. While its applications in food processing, animal feed, and renewable energy have been widely discussed, its capacity to address antibiotic resistance—especially in relation to *S. aureus*—remains underexplored and merits systematic investigation. Preliminary studies have indicated that ethanolic extracts of *C. esculentus* contain flavonoids, phenolics, terpenoids, and saponin analogs [11], compounds known to exert antimicrobial effects through multiple mechanisms: compromising bacterial membrane integrity, inhibiting enzymatic function, interfering with DNA synthesis, and preventing biofilm formation [12,13]. However, a comprehensive evaluation of the antimicrobial potential of different plant parts, and an in-depth analysis of possible synergistic or antagonistic interactions among these extracts, are still lacking. Addressing these knowledge gaps may unlock new pathways for the development of effective, natural antimicrobials that serve not only as alternatives to antibiotics in livestock but also as functional agents for enhancing food safety and protecting human health.

*C. esculentus*, rich in bioactive components, has garnered attention for its potential antibacterial properties; however, the interactions between components in multi-component plant extracts and their metabolic mechanisms against drug-resistant bacteria remain elusive [14,15]. This study aims to address this gap by systematically evaluating the in vitro antibacterial activity of *C. esculentus* tubers, stems–leaves and their composite extracts, via the integration of phenotypic experiments and untargeted metabolomics [16,17,18]. We hypothesize that the composite extract exerts stronger antibacterial activity against *S. aureus* than single-part extracts, with its mechanism of action involving not only cell membrane disruption but also the interference of metabolic pathways. Ultimately, this study seeks to identify the key metabolic pathways affected by the extracts, provide a theoretical foundation for optimizing plant-derived antibacterial agents targeting drug-resistant bacteria, and offer application potential in the fields of food safety, veterinary medicine, and infection control.

## 2. Materials and Methods

### 2.1. Materials and Reagents

In this study, Zhongyousha No.2 of *C. esculentus* variety was selected as the experimental material, and the seed source was provided by the Third Production Company of the Fifty-fourth Regiment, Tumushuke City, Xinjiang (geographic coordinates: 39.867316° N, 79.077980° E, elevation 2063 m, with a temperate continental arid climate). The experimental field was planted in a standardized planting pattern, with row spacing of 40 cm and plant spacing of 20 cm for hole sowing, and two seeds were planted in each hole [18,19]. *Staphylococcus aureus* (ATCC 6538) was purchased from China Institute of Veterinary Drug Control, China Center for the Preservation and Management of Veterinary Biological Strains.

The main experimental materials required for the experiment include LB nutrient broth medium, agar powder, Oxford cup (inner diameter 6 mm), sterile culture dish (diameter 90 mm), anhydrous ethanol (analytical purity), 50 mL centrifuge tube, glutaraldehyde solution (25% *v*/*v*), phosphate-buffered solution (PBS, 0.1 M, pH 7.4), high-grade pure formic acid, chromatographically pure acetonitrile, formic acid (analytical purity). All reagents were purchased from Solarbio Science & Technology Co., Ltd. (Beijing, China).

### 2.2. Extracting Compounds from Different Parts of C. esculentus

In this study, we used ethanol to extract the active components from different tissues of *C. esculentus*. The specific operation was as follows: fresh plant parts were air-dried and crushed to powder prior to extraction. Tuber tissue, stems and leaf samples were selected at an equal mass ratio mixed samples were selected, respectively [20], 50% (*v*/*v*) ethanol aqueous solution was used as the extraction solvent, the ratio of solid to liquid was 1:15 (g/mL), and the clear extract was obtained after ultrasonic treatment at 50 °C for 30 min, centrifugation at 4000× *g* for 15 min, and filtration through a 0.22 μm filter membrane [21]. The extracts post-ultrasonic treatment were used directly in all assays. The antibacterial activity of the extracts from different parts of *C. esculentus* was evaluated by adding amounts (50, 70, 150, and 250 μL).

### 2.3. Bacterial Strains and Culture Conditions

Thaw the glycerol cryopreservation tube of strain stored at −80 °C on ice, inoculate 50 μL of bacterial suspension into 10 mL of preheated LB liquid medium (pH 7.2), and place it on a constant temperature shaker (37 °C, 180 rpm) for dynamic culture for 6 h to the middle logarithmic phase (Optical Density at 600 nm = 0.6) [22]. The four-zone streaking method was used for purification of the colonies. The plate was inverted and incubated in a biochemical incubator at 37 °C for 18 h [23]. Typical colonies (round bulges, 2.0–2.5 mm in diameter, with regular edges and moist and shiny surfaces) were selected and inoculated into 5 mL of fresh LB broth and pre-incubated at 37 °C and 180 rpm for 12 h to prepare a bacterial suspension [24]. A total of 500 μL of bacterial suspension was transferred to 50 mL of LB medium for expansion culture, and the concentration of the bacterial solution was monitored in real time using spectrophotometry. When the OD_600_ value reached 1.5, the bacteria were collected by centrifugation at 5000× *g* for 10 min, washed twice with sterile physiological saline, resuspended [25], and adjusted to a final concentration of approximately 3.75 × 10^6^ CFU/mL for later use (cell density obtained via serial dilution and colony counting, seeded by spread-plating 100 μL). All operations were carried out under sterile conditions, and the medium was autoclaved at 121 °C for 20 min before use.

### 2.4. Oxford Cup Test

Fifteen milliliters of autoclaved LB agar medium (55–60 °C) was poured in measured volumes (15 mL) into sterile culture dishes (Φ90 mm) to form a uniform plane after solidification at room temperature. The standardized bacterial suspension (3.75 × 10^6^ CFU/mL) was spread evenly on the agar surface using a sterile applicator and allowed to stand for 15 min until the bacterial suspension was fully adsorbed. Four sterile Oxford cups (inner diameter 6 mm) were placed in a square array in the central area of the culture dish [26], and different amounts of *C. esculentus* tuber extract (50, 70, 150, and 250 μL) were quantitatively injected. After incubation at 37 °C for 24 h, the diameter of the inhibition zone was measured using digital calipers (accuracy 0.01 mm). Grading criteria were established according to CLSI M100 guidelines: zone diameter <10.0 mm is considered resistant (R), 10.0–14.9 mm is moderately sensitive (MS), and ≥15.0 mm is highly sensitive (HS) [27,28]. The other treatment groups were subjected to the same experimental protocol. Three technical replicates were performed for each group. The experimental data were analyzed using SPSS 26.0 software (α = 0.05). The results are expressed as mean ± standard error.

### 2.5. Scanning Electron Microscopic Observation

Bacterial suspension in the exponential growth stage (3.75 × 10^6^ CFU/mL) (20 μL) and extract solution of different treatments (stems, tubers, and mixture of equal proportions) (500 μL) were inoculated into 20 mL LB liquid medium and cultured at 37 °C and 180 rpm for 18 h. 1.5 mL of culture was collected by pre-cooling centrifugation (4 °C, 10,000× *g*, 10 min).

Samples after centrifugation were fixed with precooled 2.5% (*v*/*v*) glutaraldehyde solution (0.1M PBS, pH 7.2) at 4 °C for 12 h, washed with buffer solution of the same concentration three times (15 min each, 25 °C), and then subjected to gradient ethanol dehydration (30–100% concentration gradient, concentration interval 20%, each stage treatment for 20 min, 4 °C) [29]. Dehydrated samples were processed using a Leica EM CPD300 critical point desiccator (Wetzlar, Germany), fixed on an aluminum sample stage with carbon conductive gel, and plated with a platinum film (thickness 15 nm) using a Hitachi E-1045 ion sputtering apparatus (Tokyo, Japan) [30]. The experimental parameters of the stem–leaf and compound extract groups were consistent with those of this group, and each group contained five technical replicates.

### 2.6. Determination of the Bacterial Growth Curve

Several sterilized large test tubes were selected and 12 sequential test points were marked (0, 1.5, 3, 4, 6, 8, 10, 12, 14, 16, 20, and 24 h). Three replicates were set for each treatment group (*S. aureus*-containing extract) and control group (*S. aureus* without extract).

First, prepare LB medium. In the treatment group, 200 μL of bacterial suspension and 5 mL of *C. esculenta* extract mixture from the optimal part demonstrating bacteriostatic effects were added to 200 mL of LB medium using a sterile pipette. The mixture was thoroughly mixed, and then 5 mL was aliquoted into appropriately labeled sterile test tubes. The test tubes of the treatment and control groups were simultaneously placed in a constant-temperature shaking incubator (250 rpm) at 37 °C for dynamic culture. Samples were taken immediately at predetermined time points, absorbance values at OD_600_ were measured using a spectrophotometer, and uninoculated LB liquid medium was used as a reference solution for instrument zeroing [31].

### 2.7. Metabolomics Analysis

Samples with optimal antibacterial activity were selected based on growth curves, and 2 mL of each sample was transferred to cryovials. The samples were immediately flash-frozen in liquid nitrogen and stored long-term at −80 °C until further analysis. The frozen samples consisted of two groups: (1) *S. aureus* suspension (Sau) and (2) *S. aureus* suspension mixed with *C. esculentus* (Sau C), with six biological replicates per group. For metabolite extraction, frozen samples were first thawed, and 400 μL of precooled acetonitrile-methanol mixture (1:1, *v*/*v*) was added to each sample. Samples were thoroughly homogenized with an automatic homogenizer for 30 s, followed by cold sonication (10 min/cycle, 3 cycles total); after each cycle, samples were vortexed for 30 s to ensure sufficient mixing. The mixtures were then incubated at −20 °C for 60 min to fully precipitate impurities, followed by centrifugation at 10,000× *g* for 15 min at 4 °C. Finally, 200 μL of the supernatant was collected for metabolomic analyses. Untargeted metabolomics analysis was performed on a liquid chromatography–high-resolution mass spectrometry (LC-MS) platform, while targeted validation and quantification of target compounds were conducted using an Agilent 6495 (Santa Clara, CA, USA) liquid chromatography–triple quadrupole mass spectrometry system (LC-MS/MS). After data acquisition, raw LC-MS data were processed using the metabolomics software Progenesis QI v3.0 (Waters Corporation, Milford, MA, USA). Metabolite structural identification was achieved by matching the acquired mass spectral data to entries in the public metabolite databases HMDB (Human Metabolome Database) and Metlin.

### 2.8. Analysis of the Main Bacteriostatic Components in the Extract of C. esculentus

Tubers, stems, and leaves were collected, and the dirt on the surface was removed. After air drying, the tubers, stems, and leaves were crushed using a stirrer for 10 min to obtain powder samples. Weigh 0.5 g of the *C. esculentus* tuber powder sample into a 2 mL centrifuge tube, and a grinding bead with a diameter of 6 mm was added. Subsequently, 400 μL of extract solution (methanol-water mixture containing 0.02 mg/mL L-2-chlorophenylalanine internal standard, volume ratio 4:1) was added [32]. The samples were placed in a frozen tissue grinder and ground for 6 min at −10 °C and 50 Hz. After grinding, the samples were ultrasonically extracted at 5 °C and 40 KHz for 30 min. The extract was frozen at −20 °C for 30 min and centrifuged at 4 °C for 15 min under 13,000× *g* centrifugal force. Finally, the supernatant was collected for instrumental analysis [33]. Agilent 6495 LC-MS/MS, positive/negative ESI modes, *m*/*z* 50–1500, and database criteria (score >80% match in HMDB/Metlin), providing precise methodology for replication.

### 2.9. Quantitative Determination of Compounds

Diosmetin and naringenin were individually dissolved in 0.5 mL of analytical grade methanol to prepare 20 mg/mL stock solutions, whereas acacetin and silybin A were each dissolved in the same volume of analytical grade methanol to yield 40 mg/mL stock solutions. For the stem and leaf extract of *C. esculentus*, 2.0 mL aliquots were centrifuged for 10 min, and the resulting supernatant was collected for downstream analysis. Prior to quantitative analysis, the four flavonoids (diosmetin, naringenin, acacetin, and silybin A) were individually diluted with analytical grade methanol to a working concentration of 20 μg/mL.

### 2.10. Statistics and Analysis

Excel was used to sort and count the experimental data. SPSS software (version 23.0) was used to evaluate the statistical differences in the inhibition zone diameter and absorbance values among different treatment groups. Finally, the obtained metabolite-related datasets were uploaded to the Majorbio Cloud platform (cloud.majorbio.com) for subsequent bioinformatic analysis.

## 3. Results

### 3.1. Inhibitory Zones of S. aureus by Extracts from Different Parts of C. esculentus

**Figure 1:**
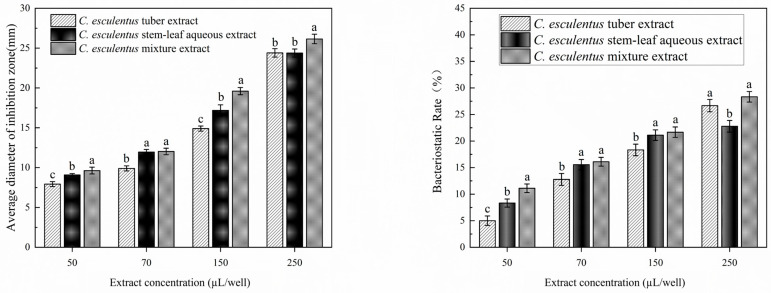
Inhibition zone measurement of different parts (extracts) of *C*. *esculentus* against *S*. *aureus*. Different lowercase letters (a–c) in the figure indicate statistically significant differences among the different ex-tract-treated groups at the same concentration (*p* < 0.05); the same lowercase letters indicate no statistically significant differences between groups.

As shown in Figure 1, extracts of tubers, stems-leaves and mixtures of *C. esculentus* showed dose-dependent bacteriostatic activity against *S. aureus*. When the volume of extract was increased from 50 μL to 250 μL, the diameter of inhibition zone of all groups increased significantly (*p* < 0.05): tuber extract increased from 7.93 mm (resistant, R) to 24.40 mm (sensitive, S); stem-leaf extract increased from 9.08 mm (R) to 24.38 mm (S); mixture extract increased from 9.63 ± 0.43 mm (R) to 26.15 mm (S). At the same volume of treatment (e.g., 250 μL), the diameter of the inhibition zone of the mixture extract (26.15 mm) increased by 7.2% and 7.3% compared with the tuber (24.40 mm) and stem (24.38 mm), respectively (*p* < 0.05). Notably, the mixture extract had already achieved a sensitive effect (19.60 mm) at 150 μL, whereas the tuber extract remained moderately sensitive (14.90 mm) at the same volume. Based on the optimal antibacterial activity exhibited by the 250 μL extract of the mixture, this result will be further explored as the core study object. The antibacterial rates of all three differently treated tiger nut extracts increased with higher addition levels. At the same concentration, the mixed extract showed the strongest antibacterial effect, followed by the tuber extract, while the stem-and-leaf extract was slightly weaker.

As shown in Table 1, the minimum inhibitory concentration (MIC) values and corresponding interpretive categories of different treatments against the target microorganism were determined as follows: vancomycin had a MIC of ≤2 μg/mL (susceptible); the solvent control had a MIC of ≥32 μg/mL (resistant); and the mixed extract of *C. esculentus* had a MIC of 8 μg/mL (intermediate).

### 3.2. Observation of the Influence of Different Extracts of C. esculentus on S. aureus by Scanning Electron Microscope

**Figure 2:**
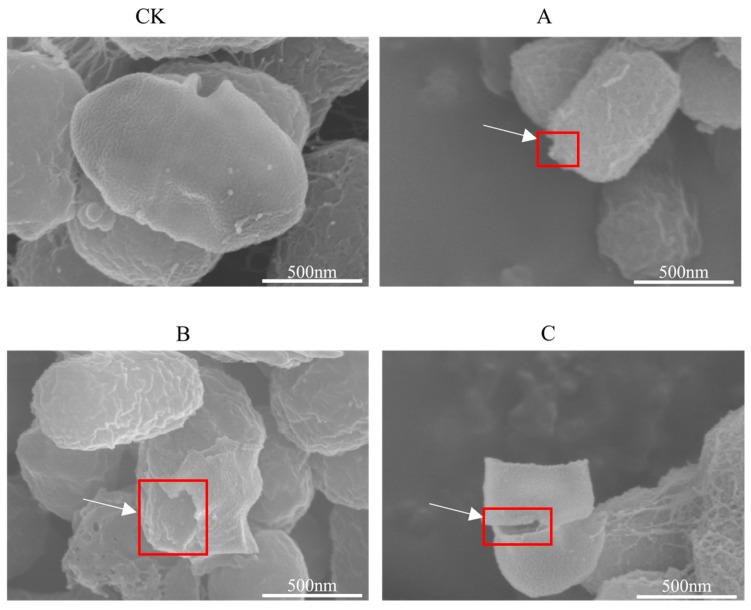
Scanning electron microscopic observation of morphological changes in *Staphylococcus aureus* treated with *C. esculentus* extract. CK is control group. (**A**) refers to treated the extract solution from tuber of *C. esculentus*, (**B**) refers to treated the extract solution from the stems-leaves of *C. esculentus*, and (**C**) refers to treated the extract solution from the mixtured (tuber, stems and leaves) of *C. esculentus*. The red box indicates the damaged part of the bacteria.

Based on the observation results of the scanning electron microscope (Figure 2), the extracts of different parts of *C. esculentus* aromatica had significant damage effects on the morphology of *S. aureus*, and the damage degree presented obvious gradient differences. Adding tuber extract (Figure 2A) resulted in slight damage: bacteria maintained a typical grape-like arrangement, the length did not change significantly, but the whole showed slight shrinkage, and the cell wall showed slight local damage. Medium damage was induced; most bacteria were deformed, the surface of most bacteria was moderately damaged, and the integrity of the cell wall was obviously damaged (Figure 2B). In contrast, the mixture extract treatment group (Figure 2C) showed the apparent rupture in cell walls in some cells: the majority of cells were damaged overall, approximately 1/3 of the bacterial tip structure was completely disintegrated, and the cell wall integrity was damaged. These results indicate that the *C. esculentus* extract can effectively destroy the cellular structural integrity of *S. aureus*, and the mixture extract exhibits the strongest antibacterial activity through action. Flavonoids destabilize membrane integrity and suppress peptidoglycan synthesis; phenolic compounds bind to proteins and inhibit enzymatic activity; terpenoids alter membrane fluidity and block DNA replication by inhibiting DNA gyrase and topoisomerase IV.

### 3.3. Growth Curve

**Figure 3:**
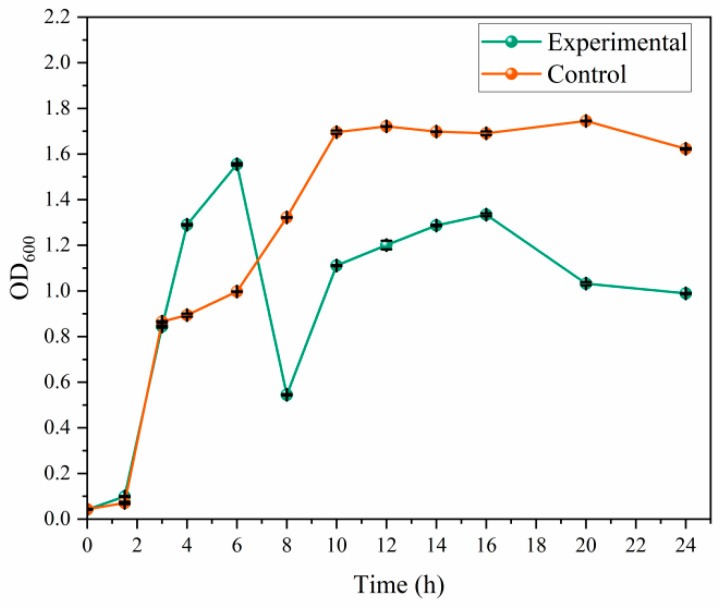
Growth curve of *S aureus* under the intervention of *C. esculentus* extract The experimental group is *S aureus* treated with *C. esculentus* mixture extract, and the control group is *S aureus*. Data were expressed as mean ± standard deviation (*n* = 3).

In order to investigate the inhibitory effect of *C. esculentus* on the growth of *S aureus*, the growth curve was measured. According to the growth curve analysis (Figure 3), *S aureus* is in the growth delay period of 0–1.5 h, enters a stable period of about 10 h, and the cell density shows a downward trend at 20 h. Adding 250 μL of the *C. esculentus* mixture extract showed significant antibacterial activity against *S. aureus*. The OD value of the bacterial suspension in the experimental group was higher than that in the control group without adding the extract solution 8 h before culture, which may be related to the temporary stimulation of bacterial metabolism in the initial stage of the extract solution. However, the OD value of the experimental group decreased continuously and was significantly lower than that of the control group after 8 h, indicating that the bacteriostatic effect of the extract solution began at this time point. When the culture time was extended to 20 h, the difference in OD_600_ values between the experimental and control groups continued to expand, confirming that the antibacterial effect had time-dependent enhancement characteristics.

The elevation of OD_600_ values within 2–6 h may reflect initial metabolic adaptation (e.g., stress responses), whereas the decline after 8 h indicates cumulative membrane damage. This observation is corroborated by SEM data and relevant literature [34]. This phenomenon is consistent with the physiological performance of bacteria under sublethal antimicrobial stress.

The above results clearly showed that the extract of the *C. esculentus* mixture had a continuously enhanced antibacterial effect on *S aureus*, and its antibacterial efficiency increased significantly with the extension of action time.

### 3.4. Analysis of the Effect of Adding the Extract of the Mixture of C. esculentus on the Metabolite of S. aureus

**Figure 4:**
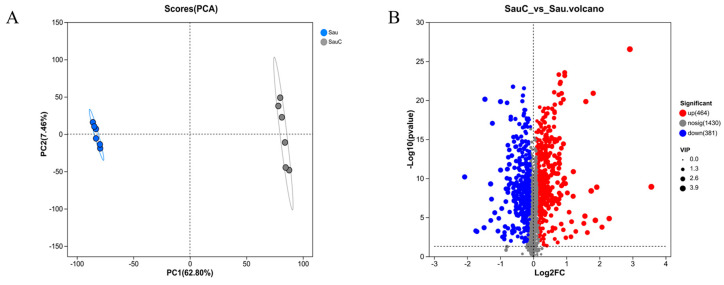
Principal component analysis (PCA, (**A**)) and volcano plot analysis (**B**) of *S. aureus* metabolome after *C. esculentus* extract treatment.

Principal Component Analysis (PCA) showed significant separation in metabolite composition between *S aureus* samples spiked with the *C. esculentus* mixture extract (Sau c) and those not spiked with the extract (Sau) (Figure 4A).

Based on the volcanic plot analysis results (Figure 4B), metabolomic analysis showed significant differences in expression after *S. aureus* was treated with the *C. esculentus* mixture extract. Among 2275 qualitative differential metabolites, a total of 845 differential metabolites (464 upregulated and 381 downregulated metabolites) were identified, accounting for 37.15% (845/2275) of the total metabolites detected. These metabolites may be involved in regulatory processes as key biomarkers.

**Figure 5:**
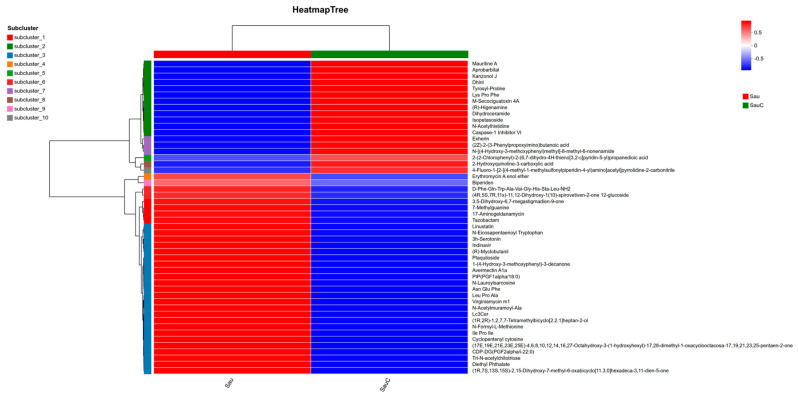
Hierarchical clustering heatmap of differentially expressed metabolites in *S. aureus* treated with *C. esculentus*.

The thermogram (Figure 5) showed that the relative expression levels of allyl isoprobarbital, tyrosylproline, (R)-norbinderine, dihydroceramide, isobatoside, N-acetylhistidine, and 2-hydroxyquinoline-3-carboxylic acid were significantly upregulated (positive correlation) in the Sau C treatment group, while the above metabolites were negatively correlated in the Sau group; in contrast, the expression levels of 7-methylguanine, 17-aminogeldanamycin, linodisaccharopicroside, N-eicosapentaenoyltryptophan, 3H-serotonin, (R)-myclobutanil, protopteridine, 1-(4-hydroxy-3-methoxyphenyl)-3-decanone, N-lauroylsarcosine, virginiamycin M1, N-acetyl-myristoyl-alanine, and cyclopentenylcytosine were significantly increased in the Sau treatment group (positive correlation), but negatively correlated in the Sau For example, the virulence-related metabolite protofern was significantly downregulated in the Sau C group, whereas the membrane structural metabolite N-lauroyl sarcosine was significantly expressed differently, indicating that the extract exerts antibacterial effects by inhibiting virulence pathways and remodeling cell membrane integrity. These results systematically explain the specific regulation of the metabolic pathway of *S. aureus* by the extract of the mixture of *C. esculentus* at the metabolic level.

**Figure 6:**
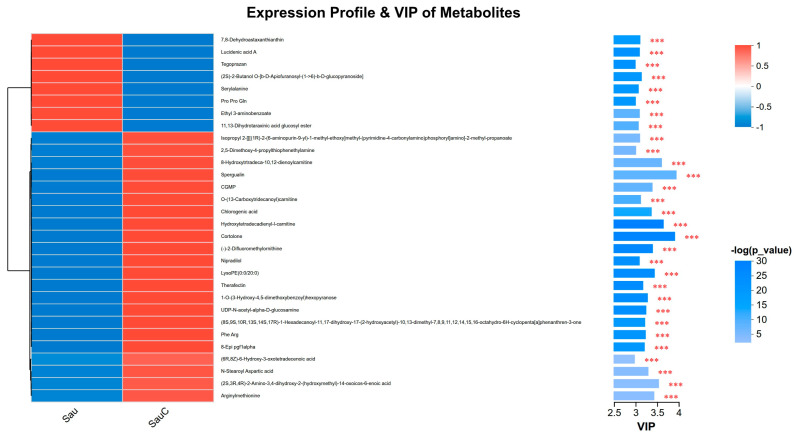
Expression profiles and variable importance in projection (VIP) values of differential metabolites in *S. aureus* treated with *C. esculentus* extract. Asterisks indicate significance levels: *** *p* < 0.001.

Based on the results of the heat map and variable importance projection (VIP) analysis (Figure 6), the metabolic profile of *S. aureus* treated with the *C. esculentus* mixture extract showed a clear trend of intergroup segregation. Red and blue areas indicate significant positive and negative correlations between the metabolites and the treatment group (Sau C), respectively. Correlation analysis showed that there was a significant positive correlation between the expression of 8-Hydroxytrtradeca-10,12-dienoylcarnitine, spergualin, cortolone, hydroxytetradecadienyl-l-carnitine and Sau C groups, with the highest VIP value and the highest statistical significance (*p* < 0.001), indicating that the difference in expression of these two metabolites was most significant between the experimental group (Sau C) and the control group (Sau).

Bar chart analysis revealed a hierarchical relationship between metabolic pathway classification and compound quantity distribution. Amino acid metabolism pathways exhibited the highest compound abundance, followed closely by lipid metabolism; collectively these constituted the core metabolic system. As a secondary metabolic cluster, xenobiotics biodegradation/metabolism and nucleotide metabolism pathways showed compound quantities at approximately 40% of the core pathways. Membrane transport pathways showed significantly lower compound counts than core metabolic pathways. Crucially, the Metabolism category dominated the classification system: 10 of 19 pathways belonged to this class, with total compound content substantially exceeding other functional categories. Environmental Information Processing showed moderate distribution levels but exhibited significant heterogeneity in glycan biosynthesis/metabolism and signal transduction pathways. Conversely, disease-related, genetic information, and cellular process pathways displayed systematically reduced compound abundance. Both disease and genetic processing pathways consistently demonstrated the lowest distribution ranges.

**Figure 7:**
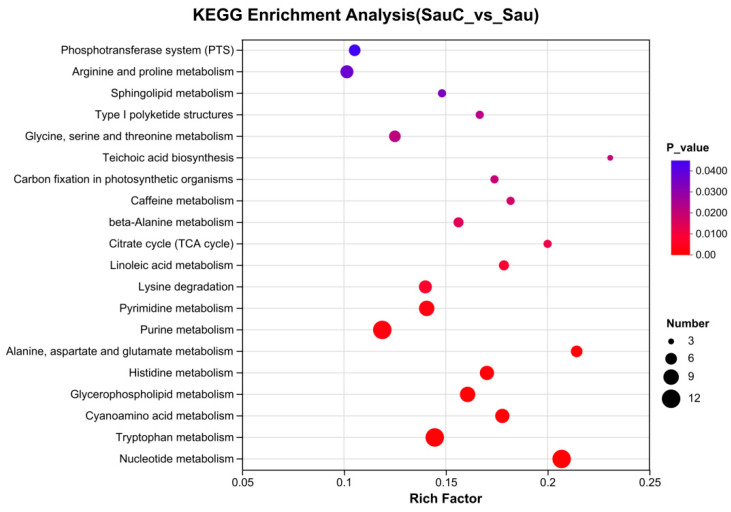
KEGG pathway topology analysis and enrichment analysis of differential metabolites in *Staphylococcus aureus* (SauC: *C. esculentus* extract-treated group; Sau: control group). Abscissa is enrichment ratio, ordinate is KEGG pathway. The size of the bubble in the figure represents how much compound is enriched in the metabolic concentration in this pathway, and the color of the bubble represents the size of the *p*-value for different enrichment significance.

Topological analysis further revealed the core node attributes of nucleotide metabolism (Figure 7). Although the Phosphotransferase system (PTS) pathway involves a limited number of compounds, it exhibits a relatively high Rich Factor and a notably significant *p*_value. This finding suggests that the PTS pathway may exert a key regulatory role in the comparison between the two groups. In contrast, the Nucleotide metabolism pathway is associated with a large number of compounds, displays a moderate Rich Factor, and has a statistically significant *p*_value—indicating that nucleotide metabolism-related processes may play an important role in the differential phenotypes between SauC and Sau.

### 3.5. Main Antibacterial Compounds in C. esculentus Extract

**Figure 8:**
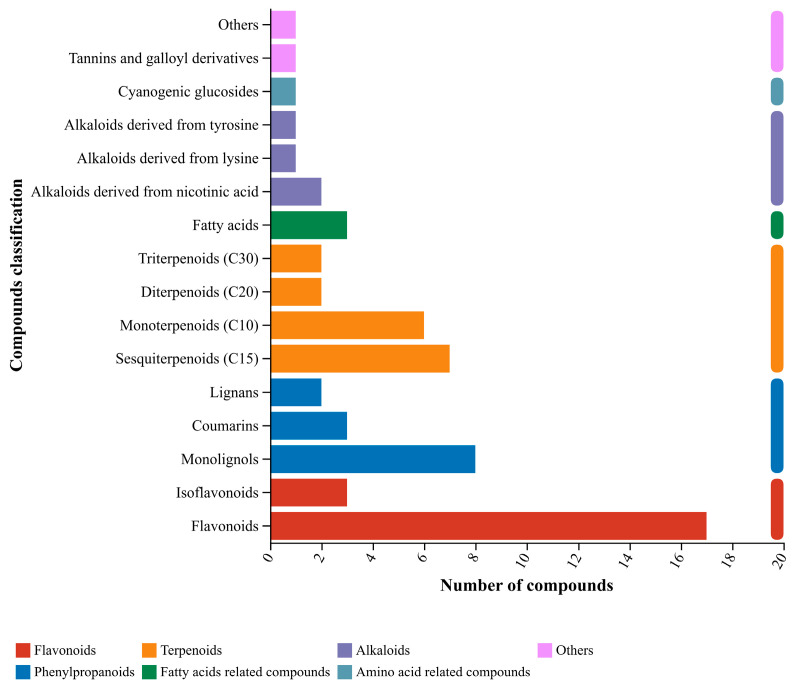
KEGG compond classification.

The KEGG pathways of *C. esculentus* illustrated in Figure 8 encompass sesquiterpenoids (C15), monoterpenoids (C10), diterpenoids (C20), lignin monomer alcohols, lignans, coumarins, tannins, galloyl derivatives, isoflavonoids, flavonoids, fatty acids, cyanogenic glucosides, and alkaloids derived from tyrosine, niacin, or lysine. Among these metabolites, flavonoids and isoflavonoids (characterized by a yellow hue) are the most abundant.

Based on the classification analysis of KEGG compounds, phenylpropanoids and flavonoids (Table 2) constitute the main components of the metabolites of *C. esculentus*, terpenoids such as diterpenes (C20), monoterpenes (C10), and sesquiterpenes (C15) are moderately abundant, while alkaloids derived from tyrosine, lysine, and nicotinic acid are relatively low.

**Figure 9:**
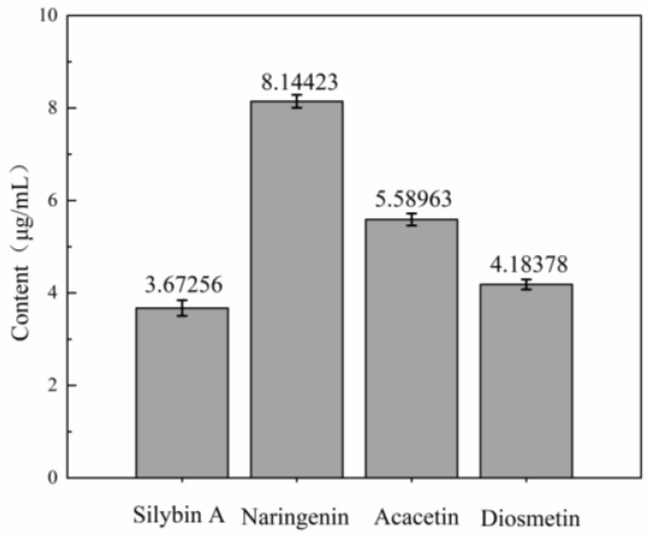
The contents of naringenin, diosmetin, acacetin, and silybin A in the extract of *C. esculentus*.

Figure 9 shows the contents of naringenin, diosmetin, acacetin, and silybin A in the *C. extract* as 8.1442 μg/mL, 5.5896 μg/mL, 4.1838 μg/mL, and 3.6726 μg/mL, respectively.

## 4. Discussion

The most striking result of this study was the superior antibacterial activity exhibited by the mixed extract of *C. esculentus*, which outperformed the individual tuber or stem-leaf extracts. The combination of compounds from different plant parts likely works synergistically, enhancing their overall antibacterial efficacy. The dose-dependent increase in inhibition zone diameters with escalating extract concentrations, particularly in the mixed extract, suggests that the antimicrobial compounds present in *C. esculentus* may interact in a complementary manner, amplifying their collective effect on *S. aureus*. This action is likely due to the diverse bioactive compounds, such as flavonoids and terpenoids. As Shukla et al. [35] describe, many bioactive small molecules derived from plants, including phenols, flavonoids, terpenoids, and alkaloids, have a wide and diverse range of pharmacological activities. They were known to disrupt bacterial membranes, inhibit DNA replication, and interfere with key enzymes involved in cellular processes. Scanning electron microscopy revealed significant morphological damage to *S. aureus* cells treated with the mixed extract, with the most extensive cellular injury observed in the mixture group. This supports the hypothesis that the extract compromises the structural integrity of bacterial cells, particularly affecting the cell wall and membrane, which are essential for maintaining cellular function and integrity [36]. Furthermore, growth curve analysis indicated a time-dependent enhancement of antibacterial effects, with the extract significantly reducing bacterial growth after 8 h of treatment. Compared with Shetty, Y. S. et al. [37] 2–6 h is the best bacteriostatic window for guava extract, and the bacteriostatic time is longer than that after delaying to 24 h when the activity decreases significantly. This temporal effect suggests that *C. esculentus* extracts may exert a sustained antibacterial influence, making them a promising candidate for controlling bacterial infections over prolonged periods.

Metabolomic analysis uncovered key changes in bacterial metabolism, including the downregulation of nucleotide synthesis and the upregulation of specific metabolic pathways such as amino acid metabolism. These metabolic shifts provide insight into the action of *C. esculentus* extracts, indicating their ability to perturb multiple cellular systems concurrently. The inhibition of glycolytic enzymes and nucleotide biosynthesis, coupled with changes in lipid metabolism, suggests a comprehensive antimicrobial mechanism that could prevent the bacterium from adapting to the antimicrobial pressure [38].

The findings of this study are consistent with previous research that has identified antimicrobial properties in various plant extracts, including those from *C. esculentus* [39,40]. Similar to earlier studies on plant-derived bioactive compounds, our results show that flavonoids, phenolic acids, and terpenoids contribute to antibacterial activity by disrupting bacterial membranes and interfering with cellular functions. However, the novel aspect of our study lies in the effect of extracts from different parts of the plant, which has not been extensively explored in the literature. Moreover, while some studies have reported on the antibacterial activity of *C. esculentus* extracts [40], our comprehensive analysis using both phenotypic assays and metabolomic profiling provides new insights into the mechanistic underpinnings of the observed effects, highlighting the plant’s potential as a natural antimicrobial agent against resistant pathogens like *S. aureus*.

Based on VIP analysis, the levels of spergualin were markedly elevated in the test group in comparison to the control group. Spergualin, a natural polyamine antibiotic, exhibits a wide-ranging inhibitory effect against both Gram-positive and Gram-negative bacteria [41]. Umezawa et al. [42] demonstrated the significant antibacterial activity of spergualin. Yang et al. [43] identified the spergualin synthesis gene cluster in the genome through secondary metabolite analysis. Both *S. aureus* and *Escherichia coli* tested positive for the presence of this gene cluster. It is suggested that spergualin is related to the antibacterial activity of *C. esculentus*.

As illustrated in Figure 9, the contents of these components exhibit a direct correlation with the observed antibacterial activity. As the most abundant compound in the extract, naringenin likely contributes predominantly to its antibacterial efficacy, as prior studies have confirmed its capacity to disrupt bacterial membrane integrity and inhibit nucleic acid synthesis. Diosmetin and acacetin, the second most abundant compounds, are well-documented to interfere with bacterial enzymatic activities (e.g., key glycolytic enzymes), which aligns with the glycolytic pathway inhibition detected in our metabolomic analysis. Despite its relatively low concentration, silybin A may enhance the potency of other bioactive components via synergistic interactions, such as potentiating the disruption of bacterial cell wall synthesis—consistent with the bacterial morphological damage visualized by scanning electron microscopy (SEM). This multi-component mechanism mitigates the risk of bacteria acquiring drug resistance via a single mutation, thereby underscoring the superiority of the mixed *C. esculentus*.

These results are consistent with previous studies on the antimicrobial properties of *C. esculentus*, in which ethanolic and acetone extracts inhibited the growth of *S. aureus*, an effect attributed to similar classes of bioactive compounds [44]. However, inconsistencies have been reported. For instance, one study observed no antibacterial activity of ethanolic extracts against *S. aureus*, which may be attributed to differences in extraction solvents, plant varieties, or bacterial strains. Our study addresses this gap by focusing on mixed extracts, demonstrating that their enhanced antibacterial potency results from synergistic effects, an area that has been underexplored. Importantly, while phenotypic assays confirmed the inhibitory effect, the integration of metabolomics in our work provides further mechanistic insights, revealing a broader disruptive impact beyond mere membrane damage [45].

Compared with previously reported mono-part or mono-compound preparations, the mixed tuber–leaf ethanol extract of *C. esculentus* evaluated herein yielded a 26.15 mm inhibition zone against *S. aureus*—approximately 30% larger than the 19.3 mm recorded for leaf-only flavonoids [44] and still 15% superior to the high-flavonoid *Adhatoda vasica* extract (22 mm) [35]. SEM evidence of complete tip disintegration in one-third of the cells has not been documented for either single-part tiger-nut extracts [45] or purified monoterpenoids such as myrtenol [13].

From a biological perspective, these findings suggest that *C. esculentus* extracts could function as multi-target agents. The likelihood of resistance emergence is reduced compared to single-mechanism antibiotics because simultaneous mutations in multiple pathways would be required for bacterial evasion. This multi-target approach is particularly relevant for combating *S. aureus* in veterinary medicine and food safety, where antibiotic overuse is a major driver of resistance. Potential applications include the development of botanical formulations for topical use or as feed additives, which could help mitigate infections in livestock or preserve food without relying on synthetic preservatives. However, the observed metabolic shifts, such as the upregulation of amino acid metabolism, might promote the formation of persister cells under sublethal stress. This phenomenon, similar to the formation of small colony variants, could potentially prolong infections if the bacterial population is not completely eradicated [46,47].

This research contributes to the field of natural antimicrobials by demonstrating the efficacy of *C. esculentus* extracts against resistant pathogens. The mechanistic insights provided pave the way for the targeted isolation of bioactive compounds. Nonetheless, this study has limitations. The in vitro model does not account for in vivo complexities such as bioavailability and host–pathogen interactions. Furthermore, the limited diversity of bacterial strains tested may not represent the full spectrum of clinical variability. Future studies should focus on validating these findings in animal models, utilizing proteomics to confirm putative enzyme targets, and investigating potential synergies with conventional antibiotics to improve therapeutic efficacy.

## 5. Conclusions

This study demonstrated significant antibacterial effects of the mixed *C. esculentus* extract against multi-drug resistant *S. aureus*. Its efficacy exceeded that of individual extracts. Metabolomics studies revealed that mixed extract intervention inhibited the nucleotide biosynthesis pathway in *S. aureus*, reshaped lipid metabolism, and disrupted amino acid pathways. These alterations caused bacterial growth stagnation by suppressing energy metabolism and inducing membrane dysfunction. Active component analysis identified four flavonoids—acacetin, diosmetin, naringenin, and silybin A—as critical antibacterial substances, offering a novel plant-derived solution for combating *S. aureus*.

## Figures and Tables

**Table 1:** MIC Values and Antimicrobial Susceptibility Categories of Each Treatment Group.

Treatment	MIC (μg/mL)	Interpretive Category
Vancomycin	≤2	Susceptible
Solvent	≥32	Resistant
*C. esculentus* mixture extract	8	Intermediate

**Table 2:** Identification and characterization of flavonoid compounds in *C. esculentus* extracts.

Name	Mode	Formula	HMDB Superclass	Compound Classification
Acacetin	Positive ion	C_16_H_12_O_5_	Phenylpropanoids and polyketides	Flavonoids
Naringenin	Positive ion	C_15_H_12_O_5_	Phenylpropanoids and polyketides	Flavonoids
Diosmetin	Positive ion	C_16_H_12_O_6_	Phenylpropanoids and polyketides	Flavonoids
Silybin A	Negative ions	C_25_H_22_O_10_	Phenylpropanoids and polyketides	Flavonoids

## Data Availability

The original contributions presented in this study are included in the article. Further inquiries can be directed to the corresponding author.

## References

[1] Pahlevi M.R., Murakami K., Hiroshima Y., Murakami A., Fujii H. (2022). pruR and PA0065 Genes Are Responsible for Decreasing Antibiotic Tolerance by Autoinducer Analog-1 (AIA-1) in Pseudomonas aeruginosa. Antibiotics.

[2] Lasek-Nesselquist E., Lu J., Schneider R., Ma Z., Russo V., Mishra S., Pai M.P., Pata J.D., McDonough K.A., Malik M. (2019). Insights Into the Evolution of *Staphylococcus aureus* Daptomycin Resistance from an in vitro Bioreactor Model. Front. Microbiol..

[3] Liu E., Chen Y., Xu J., Gu S., An N., Xin J., Wang W., Liu Z., An Q., Yi J. (2022). Platelets Inhibit Methicillin-Resistant *Staphylococcus aureus* by Inducing Hydroxyl Radical-Mediated Apoptosis-Like Cell Death. Microbiol. Spectr..

[4] Zhang J., Wang Z., Wang H.-Y., Chung C.-R., Horng J.-T., Lu J.-J., Lee T.-Y. (2022). Rapid Antibiotic Resistance Serial Prediction in *Staphylococcus aureus* Based on Large-Scale MALDI-TOF Data by Applying XGBoost in Multi-Label Learning. Front. Microbiol..

[5] Wang H., Kim S., Kim J., Park S.-D., Uh Y., Lee H. (2014). Multiplex Real-Time PCR Assay for Rapid Detection of Methicillin-Resistant Staphylococci Directly from Positive Blood Cultures. J. Clin. Microbiol..

[6] Duvauchelle V., Majdi C., Bénimélis D., Dunyach-Remy C., Meffre P., Benfodda Z. (2021). Synthesis, Structure Elucidation, Antibacterial Activities, and Synergistic Effects of Novel Juglone and Naphthazarin Derivatives Against Clinical Methicillin-Resistant *Staphylococcus aureus* Strains. Front. Chem..

[7] Guan C., Liu T., Li Q., Wang D., Zhang Y. (2022). Analyzing the Effect of Baking on the Flavor of Defatted Tiger Nut Flour by E-Tongue, E-Nose and HS-SPME-GC-MS. Foods.

[8] Yang X., Niu L., Zhang Y., Ren W., Yang C., Yang J., Xing G., Zhong X., Zhang J., Slaski J. (2022). Morpho-Agronomic and Biochemical Characterization of Accessions of Tiger Nut (*Cyperus esculentus*) Grown in the North Temperate Zone of China. Plants.

[9] Zhang Y., Sun S. (2023). Tiger nut (*Cyperus esculentus* L.) oil: A review of bioactive compounds, extraction technologies, potential hazards and applications. Food Chem. X.

[10] Zhang S., Li P., Wei Z., Cheng Y., Liu J., Yang Y., Wang Y., Mu Z. (2022). Cyperus (*Cyperus esculentus* L.): A Review of Its Compositions, Medical Efficacy, Antibacterial Activity and Allelopathic Potentials. Plants.

[11] Prakash N., Ragavan B. (2009). Phytochemical observation and antibacterial activity of *Cyperus esculentus* L. Anc. Sci. Life.

[12] Chen S., Wang X., Cheng Y., Gao H., Chen X. (2023). A Review of Classification, Biosynthesis, Biological Activities and Potential Applications of Flavonoids. Molecules.

[13] Cordeiro L., Figueiredo P., Souza H., Sousa A., Andrade-Júnior F., Barbosa-Filho J., Lima E. (2020). Antibacterial and Antibiofilm Activity of Myrtenol against *Staphylococcus aureus*. Pharmaceuticals.

[14] Barros Cota B., Batista Carneiro de Oliveira D., Carla Borges T., Cristina Catto A., Valverde Serafim C., Rogelis Aquiles Rodrigues A., Kohlhoff M., Leomar Zani C., Assunção Andrade A. (2021). Antifungal activity of extracts and purified saponins from the rhizomes of *Chamaecostus cuspidatus* against *Candida* and *Trichophyton* species. J. Appl. Microbiol..

[15] Sood S., Bhardwaj V., Mangal V., Kardile H., Dipta B., Kumar A., Singh B., Siddappa S., Sharma A.K., Dalamu (2024). Development of near homozygous lines for diploid hybrid TPS breeding in potatoes. Heliyon.

[16] Yu Y., Dong J., Wang Y., Gong X. (2021). RNA-seq analysis of antibacterial mechanism of *Cinnamomum camphora* essential oil against *Escherichia coli*. PeerJ.

[17] Ellboudy N.M., Elwakil B.H., Shaaban M.M., Olama Z.A. (2023). Cinnamon Oil-Loaded Nanoliposomes with Potent Antibacterial and Antibiofilm Activities. Molecules.

[18] Toh S.C., Lihan S., Bunya S.R., Leong S.S. (2023). In vitro antimicrobial efficacy of *Cassia alata* (Linn.) leaves, stem, and root extracts against cellulitis causative agent *Staphylococcus aureus*. BMC Complement. Med. Ther..

[19] Yang D., Liu Y., Wang Y., Gao F., Zhao J., Li Y., Li X. (2020). Effects of Soil Tillage, Management Practices, and Mulching Film Application on Soil Health and Peanut Yield in a Continuous Cropping System. Front. Microbiol..

[20] Kubica P., Szopa A., Kokotkiewicz A., Miceli N., Taviano M.F., Maugeri A., Cirmi S., Synowiec A., Gniewosz M., Elansary H.O. (2020). Production of Verbascoside, Isoverbascoside and Phenolic Acids in Callus, Suspension, and Bioreactor Cultures of Verbena officinalis and Biological Properties of Biomass Extracts. Molecules.

[21] Huang P., Xia L., Zhou L., Liu W., Wang P., Qing Z., Zeng J. (2021). Influence of different elicitors on BIA production in Macleaya cordata. Sci. Rep..

[22] Liu Y., She P., Xu L., Chen L., Li Y., Liu S., Li Z., Hussain Z., Wu Y. (2021). Antimicrobial, Antibiofilm, and Anti-persister Activities of Penfluridol Against *Staphylococcus aureus*. Front. Microbiol..

[23] Huang T., Zhou Z., Li Q., Tang X., Chen X., Ge Y., Ling J. (2021). Light-Triggered Adhesive Silk-Based Film for Effective Photodynamic Antibacterial Therapy and Rapid Hemostasis. Front. Bioeng. Biotechnol..

[24] Libis V., MacIntyre L.W., Mehmood R., Guerrero L., Ternei M.A., Antonovsky N., Burian J., Wang Z., Brady S.F. (2022). Multiplexed mobilization and expression of biosynthetic gene clusters. Nat. Commun..

[25] Hu Y., Xing Y., Ye P., Yu H., Meng X., Song Y., Wang G., Diao Y. (2023). The antibacterial activity and mechanism of imidazole chloride ionic liquids on *Staphylococcus aureus*. Front. Microbiol..

[26] Chi Y., Sun W., Zhou L., Pei S., Zeng H., Cheng Y., Chai S. (2023). The preparation of hybrid silicon quantum dots by one-step synthesis for tetracycline detection and antibacterial applications. Anal. Methods.

[27] Paschoalini B.R., Nuñez K.V.M., Maffei J.T., Langoni H., Guimarães F.F., Gebara C., Freitas N.E., Dos Santos M.V., Fidelis C.E., Kappes R. (2023). The Emergence of Antimicrobial Resistance and Virulence Characteristics in *Enterococcus* Species Isolated from Bovine Milk. Antibiotics.

[28] Sheng J.W., Liu D.M., Jing L., Xia G.X., Zhang W.F., Jiang J.R., Tang J.B. (2019). Striatisporolide A, a butenolide metabolite from Athyrium multidentatum (Doll.) Ching, as a potential antibacterial agent. Mol. Med. Rep..

[29] Zhang X.-F., Li Q.-Y., Wang M., Ma S.-Q., Zheng Y.-F., Li Y.-Q., Zhao D.-L., Zhang C.-S. (2022). 2E,4E-Decadienoic Acid, a Novel Anti-Oomycete Agent from Coculture of Bacillus subtilis and Trichoderma asperellum. Microbiol. Spectr..

[30] Santos A.L., van Venrooy A., Reed A.K., Wyderka A.M., García-López V., Alemany L.B., Oliver A., Tegos G.P., Tour J.M. (2022). Hemithioindigo-Based Visible Light-Activated Molecular Machines Kill Bacteria by Oxidative Damage. Adv. Sci..

[31] Habib G., Zhu Q., Sun B. (2018). Bioinformatics and Functional Assessment of Toxin-Antitoxin Systems in *Staphylococcus aureus*. Toxins.

[32] Xiong Q., Sun C., Shi H., Cai S., Xie H., Liu F., Zhu J. (2022). Analysis of Related Metabolites Affecting Taste Values in Rice under Different Nitrogen Fertilizer Amounts and Planting Densities. Foods.

[33] Siddaiah C., Kumar Bm A., Deepak S.A., Lateef S.S., Nagpal S., Rangappa K.S., Mohan C.D., Rangappa S., Kumar S M., Sharma M. (2020). Metabolite Profiling of *Alangium salviifolium* Bark Using Advanced LC/MS and GC/Q-TOFTechnology. Cells.

[34] Cui Y., Kim S.H., Kim H., Yeom J., Ko K., Park W., Park S. (2012). AFM probing the mechanism of synergistic effects of the green tea polyphenol (-)-epigallocatechin-3-gallate (EGCG) with cefotaxime against extended-spectrum beta-lactamase (ESBL)-producing *Escherichia coli*. PLoS ONE.

[35] Shukla S., Ahirwal L., Bajpai V.K., Huh Y.S., Han Y.K. (2017). Growth Inhibitory Effects of *Adhatoda vasica* and Its Potential at Reducing Listeria monocytogenes in Chicken Meat. Front. Microbiol..

[36] Zhang F., Graham J., Zhai T., Liu Y., Huang Z. (2022). Discovery of MurA Inhibitors as Novel Antimicrobials through an Integrated Computational and Experimental Approach. Antibiotics.

[37] Shetty Y.S., Shankarapillai R., Vivekanandan G., Shetty R.M., Reddy C.S., Reddy H., Mangalekar S.B. (2018). Evaluation of the Efficacy of Guava Extract as an Antimicrobial Agent on Periodontal Pathogens. J. Contemp. Dent. Pract..

[38] Montso P.K., Mnisi C.M., Ayangbenro A.S. (2022). Caecal microbial communities, functional diversity, and metabolic pathways in Ross 308 broiler chickens fed with diets containing different levels of Marama (*Tylosema esculentum*) bean meal. Front. Microbiol..

[39] Kani A.N., Dovi E., Aryee A.A., Han R., Qu L. (2022). Efficient removal of 2,4-D from solution using a novel antibacterial adsorbent based on tiger nut residues: Adsorption and antibacterial study. Environ. Sci. Pollut. Res. Int..

[40] Belmadani N., Kassous W., Keddar K., Amtout L., Hamed D., Douma-Bouthiba Z., Costache V., Gérard P., Ziar H. (2024). Functional *Cyperus esculentus* L. Cookies Enriched with the Probiotic Strain *Lacticaseibacillus rhamnosus* SL42. Foods.

[41] Assimon V.A., Shao H., Garneau-Tsodikova S., Gestwicki J.E. (2015). Concise Synthesis of Spergualin-Inspired Molecules with Broad-Spectrum Antibiotic Activity. MedChemComm.

[42] Umezawa H., Kondo S., Iinuma H., Kunimoto S., Ikeda Y., Iwasawa H., Ikeda D., Takeuchi T. (1981). Structure of an antitumor antibiotic, spergualin. J. Antibiot..

[43] Xu Y., Liang X., Hyun C.G. (2024). Isolation, Characterization, Genome Annotation, and Evaluation of Hyaluronidase Inhibitory Activity in Secondary Metabolites of *Brevibacillus* sp. JNUCC 41: A Comprehensive Analysis through Molecular Docking and Molecular Dynamics Simulation. Int. J. Mol. Sci..

[44] Jing S., Wang S., Li Q., Zheng L., Yue L., Fan S., Tao G. (2016). Dynamic high pressure microfluidization-assisted extraction and bioactivities of *Cyperus esculentus* (*C. esculentus* L.) leaves flavonoids. Food Chem..

[45] Wani P.A., Omobolanle L.A., Hamid B., Fayokemi R.A., Perveen K., Bukhari N.A., Sayyed R.Z., Mastinu A. (2024). Evaluation of destruction of bacterial membrane structure associated with anti-quorum sensing and ant-diabetic activity of *Cyperus esculentus* extract. Heliyon.

[46] Zhou J., He C., Yang H., Shu W., Liu Q. (2024). Integrative omics analysis reveals insights into small colony variants of *Staphylococcus aureus* induced by sulfamethoxazole-trimethoprim. BMC Microbiol..

[47] Tuchscherr L., Löffler B., Proctor R.A. (2020). Persistence of *Staphylococcus aureus*: Multiple Metabolic Pathways Impact the Expression of Virulence Factors in Small-Colony Variants (SCVs). Front. Microbiol..

